# Barriers to access to outpatient mental health care for refugees and asylum seekers in Switzerland: the therapist’s view

**DOI:** 10.1186/s12888-020-02783-x

**Published:** 2020-07-17

**Authors:** Nikolai Kiselev, Naser Morina, Matthis Schick, Birgit Watzke, Ulrich Schnyder, Monique C. Pfaltz

**Affiliations:** 1 Department of Consultation-Liaison Psychiatry and Psychosomatic Medicine, University Hospital Zurich, Zurich, Switzerland; 2 Department of Clinical Psychology and Psychotherapy Research, University of Zurich, Zurich, Switzerland; 3 Medical Faculty, University of Zurich, Zurich, Switzerland

**Keywords:** Mental health care services, Refugee mental health, Barriers, Switzerland, Asylum seekers, Interpreters

## Abstract

**Background:**

More than 120,000 refugees and asylum seekers are currently living in Switzerland. The prevalence of mental disorders among this population is significantly higher than that in the general population. While effective treatment options and cross-cultural, specialized treatment centers exist, they tend to be overloaded by their target populations. General outpatient primary health care providers might be able to compensate for the lack of specialized treatment slots. To date, however, it is unknown how often and under what conditions (e.g., length of waiting lists) refugees and asylum seekers are treated outside of specialized centers and whether there are barriers that prevent providers in outpatient settings from treating more patients in this subgroup. The present study aimed to assess the challenges and barriers faced by psychiatrists and psychotherapists working in outpatient settings in Switzerland in treating refugees and asylum seekers to determine the potential capacity of this group to provide mental health care.

**Methods:**

An online survey was conducted during the winter of 2017/2018. The survey was constructed in three official languages and took 10–15 min to complete. Spearman’s correlations, Mann-Whitney U-Tests, and Chi-squared tests were conducted to analyze the data.

**Results:**

Eight hundred and sixty-seven (*N* = 867) psychotherapists and psychiatrists working in outpatient settings completed the survey: 43% of them reported having treated between 1 and 9 refugees or asylum seekers in the past 12 months, and a further 13% reported treating 10 or more. Interpreters were used for almost every other patient with a refugee or asylum-seeker background. At the same time, the funding of interpreters, as well as the funding of treatment in general, were reported to be the biggest hurdles to treating more refugees and asylum seekers.

**Conclusions:**

Given the low number of patients rejected for capacity reasons (between 2 and 5%) and the median waiting times for the admission of new patients ranging between 2 and 3 weeks, outpatient primary mental health care providers might treat more refugees and asylum seekers and relieve specialized treatment centers. However, barriers such as lack of funding of interpreters seem to hinder them. Appropriate steps by the authorities are needed to improve the current situation.

## Background

In 2019, there were almost 80 million forcibly displaced people worldwide [1]. Among those displaced, almost 26 million people were refugees and 4.2 million asylum seekers. Due to ongoing armed conflicts, e.g., in Syria, Afghanistan, South Sudan, Myanmar, and Somalia, the source of 67% of the world’s refugees [2], it is highly unlikely that this figure will decrease in the near future. More than 126,000 refugees and asylum seekers are currently living in Switzerland [3]. The number of former refugees – persons who have left asylum and refugee structures (e.g. by naturalization) and now live in Switzerland – is unknown. However, based on Switzerland’s strong tradition of providing asylum, the number of former refugees is expected to be several times higher than the number of current refugees and asylum seekers. Over the past 20 years, at least 14,000 asylum applications were submitted annually, and over the past five years, more than 110,000 asylum seekers were registered in Switzerland [3].

### Mental health of refugees and asylum seekers

Refugees and asylum seekers are a very vulnerable group due to the high prevalence of stressful experiences before, during, and after flight [4–6]. The relationships between trauma exposure and psychological problems and between post-migration stress and mental disorders are well-established [7–12]. Among refugees and asylum seekers, self-reported symptoms of common mental disorders such as depression, anxiety, and trauma-related disorders like post-traumatic stress disorder (PTSD) are significantly higher than those in the general population [13–17]. For example, the reported prevalence rates of depression and PTSD range between 2.3 and 80% and between 4.4 and 86%, respectively [18, 19], resulting from a broad range of clinical and methodological factors that contribute to the observed heterogeneity. Overall, the reported prevalence of common mental disorders such as anxiety, depression, and PTSD in conflict-affected or displaced populations around the world might be estimated to be around 20 to 30% [20, 21]. In contrast, the one-year prevalence in the general population in Western countries ranges between 4.6 and 7.4% for depression [22] and between 3.5 and 4.7% for PTSD [23].

### Situation in Switzerland

Currently, there are no representative data on the state of mental health of refugees and asylum seekers in Switzerland. Besides studies that report the rates of particular mental health disorders among refugees and asylum seekers who are seeking treatment or are already in treatment [24–28], only a few studies have been published on this topic so far. However, they are all limited by their small sample sizes [29–35]. These studies report the prevalence of self-reported symptoms of common mental disorders among refugees and asylum seekers in Switzerland, ranging from 33 to 63% for depression, from 24 to 54% for PTSD, and from 10 to 85% for anxiety disorders [29, 30, 33, 36]. Comorbidities are high as well [37, 38], pointing to high levels of distress. Given the numbers of refugees and asylum seekers in Switzerland, we estimate that more than 30′000 of the current refugees and asylum seekers in Switzerland are affected by at least one common mental disorder.

In Switzerland, the mandatory health insurance model guarantees everyone access to qualified medical care. Due to this obligation, the costs of primary care, including general practice services as well as prescribed medication, are fully covered. Forty sessions of psychotherapy performed or referred by a psychotherapist doctor are fully covered in most cases. Additional sessions require a re-approved referral. In- and outpatient psychiatric and psychotherapeutic services are covered by mandatory insurance as long as the vital criterion of referral by a psychiatrist is met, excluding a retention fee of around 10%. If patients are unable to pay retention fees, these fees are covered by social or refugee welfare. Psychotherapy provided by psychological psychotherapists (psychologists with post-graduate training in psychotherapy) is, however, only covered if the psychotherapist works at the premises of a psychiatrist and the treatment was delegated (so-called Delegations Model) by the psychiatrist to the psychotherapist. This means that psychotherapy provided by self-employed psychological psychotherapists is not covered by insurance, and patients have to pay for such services themselves.

While effective evidence-based psychotherapeutic and psychopharmacological treatments for mental disorders exist, little is known about the access of refugees and asylum seekers to mental health care in Switzerland. A study of asylum seekers found that in Switzerland, asylum seekers were often underdiagnosed and inappropriately treated, which was – at least partially –attributable to communication difficulties [30]. It is estimated that between $$ \raisebox{1ex}{$2$}\!\left/ \!\raisebox{-1ex}{$3$}\right. $$ and ¾ of asylum seekers are affected by severe communication difficulties between the patient and health care provider [39]. Frequently, family members or friends serve as lay-interpreters [40], leading to misunderstandings, keeping details secret, and wrong diagnoses [41–43]. Trained interpreters could solve this problem in Switzerland, yet their financing is unregulated and not covered by insurance [35, 44, 45]. Limited access to trained interpreters for mental health care in Switzerland seems to be a chronic problem and was already noted 20 years ago [46]. Official reports for the Federal Office of Public Health (FOPH) as well as the State Secretariat for Migration (SEM) showed that the financing of interpreter services remains one of the key problems in the mental health care supply of refugees in Switzerland [35, 42]. However, further barriers, such as stigma of mental disorders and lack of information about mental health treatment, exist [4].

Finally, it was recently reported that specialized mental health treatment services for refugees and asylum seekers are overloaded across Switzerland [34, 35, 40]. This lack of capacity corresponds with long waitlists for specialized services [34, 35, 40]. A recent study suggests that as a result of long waiting times, many refugees and asylum seekers coordinators do not attempt to refer patients to specialized mental health care centers [40]. Long waitlists might partially feed back to the unregulated situation regarding the cost coverage of trained interpreters in Switzerland: Due to the lack of funding for interpreters, patients unable to speak the local language are usually referred to specialized “transcultural” mental health care, resulting in massive supply overload [34, 40, 44]. Yet, the previous findings (regarding lack of capacity, long waitlists, and lack of funding for interpreters) reflect primarily the experiences and opinions of Cantonal Medical Officers and Cantonal Refugees and Asylum Seekers Coordinators, specialized providers, and mental health care providers, or they are based on small samples [34, 35].

In sum, a considerable portion (up to 40%) of refugees and asylum seekers in Switzerland may suffer from common mental health problems [29, 30, 36]. Several barriers that prevent refugees and asylum seekers from accessing mental health care in Switzerland have been reported. Language problems and limited treatment capacities in specialized treatment centers resulting in long waiting lists and rejections in combination with the insufficient financing of interpreters were the most commonly reported difficulties [35, 39, 40, 42]. However, to date, there is no reliable information on the challenges and barriers faced by mental health care providers (i.e., psychiatrists and psychotherapists) working in non-specialized outpatient mental health care in Switzerland in treating refugees and asylum seekers. Yet, more detailed reporting of mental health care providers’ viewpoints and experiences in treating traumatized refugees and asylum seekers would be desirable as these professional groups would be well-suited to fill the current treatment gap, resulting from a lack of specialized treatment centers and long waiting lists.

### Aims and hypotheses

The first aim of this study was to investigate the extent to which psychiatrists and psychotherapists currently provide outpatient treatment to refugees and asylum seekers in Switzerland. Second, we aimed at assessing the circumstances under which they are providing treatment. That is, to determine whether primary mental health care providers might fill the current treatment gap, we focused on provider-reported lengths of time-to-treatment, rejection quotas for capacity reasons, and the frequency and perceived quality of use of trained vs. untrained interpreters. To fulfill these aims, we limited our observations to the past 12 months. Third, we assessed provider-perceived circumstances preventing psychiatrists and psychotherapists from treating more refugees and asylum seekers in Switzerland. Due to the previously reported deficits regarding access to mental health care, we did investigate enablers but focused on assessing barriers.

In addition to exploring psychiatrist and psychotherapist treatment rates of refugees and asylum seekers in outpatient settings in the past 12 months (aim 1), we hypothesized (regarding aim 2) that psychiatrists and psychotherapists treating more refugees and asylum seekers would (2a) report longer waiting times for the admission of new patients and (2b) higher rejection rates for capacity reasons. Given the previously reported communication difficulties [36, 39, 41–43]), we aimed to explore the frequency of use of translation services and providers’ general satisfaction with translation services. We hypothesized (2c) that the rate of use of trained/untrained interpreters would influence the providers’ general satisfaction with translation services and (2d) that satisfaction with translation would be significantly higher among providers frequently using trained interpreters than among providers using trained interpreters less frequently. Regarding aim 3, we predicted that language-related obstacles would constitute the most commonly mentioned barrier.

## Methods

### Sample

Two target populations were identified: psychiatrists (including psychiatric residents) and psychological psychotherapists (including psychological psychotherapists in training). In the following, we will use the term “psychiatrist” to refer to the first group and the term “psychotherapist” to refer to the latter group.

From the public webpage of the Swiss Medical Association (www.doctorfmh.ch) [FMH], we identified a national sample of 3561 psychiatrists. After a comprehensive search of the World Wide Web, the email addresses of 2319 psychiatrists were sourced. In addition, we obtained the email addresses of 643 psychiatric residents from the largest Swiss organization providing post-graduate education and training to residents specializing in psychiatry (Weiterbildungsverein Psychiatrie und Psychotherapie Zürich, Zentral-, Nord- und Ostschweiz (WBV)). The email addresses of 2967 psychotherapists were obtained from the Federation of Swiss Psychologists (FSP). Forty-seven sub-organizations of FSP were informed about the study and were asked to inform their members as well. Additionally, the Swiss Society for Applied Psychology (SBAP) and the Association of Swiss Psychotherapists (ASP) were asked to disseminate an invitation to their members to participate in the study. Finally, all Swiss psychiatric hospitals and institutional providers of mental health care were identified. From their websites, the email addresses of all 496 heads of department, head psychiatrists, and head psychologists were retrieved.

### Procedure

Data were collected using an online survey. On the commencement day, December 7th, 2017, each identified psychiatrist and psychotherapist (*n* = 5929) received an email with a brief project description and an invitation to participate in one of three national languages (German, French, Italian). On the same day, heads of department, head psychiatrists, and head psychologists received a personalized email from either the former head of department (US) or the head psychologist (MP) of the Department of Consultation-Liaison Psychiatry and Psychosomatics (at that time, Department of Psychiatry and Psychotherapy) at the University Hospital Zurich asking the recipients to forward the survey invitation to their employees.

To enable the inclusion of mental health care providers with and without experience in treating the population of interest, it was noted in the invitation that participants with no or minimal experience in treating refugees or asylum seekers were explicitly welcome to participate in the survey. To improve participation rates, the research team informed potential participants that 5 Swiss francs would be donated to an organization of the participant’s choice (Médecins Sans Frontières, World Wildlife Fund, or Happiness Again Malki Center^1^) for each fully completed survey. Moreover, two reminders were sent on January 9th and 30th, 2018, resulting in a significant increase in the number of completed questionnaires prior to closing the survey on February 21st, 2018.

### Online survey

The survey was published and completed online by means of the SoSci Survey software [47]. This software allowed us to collect raw data without recording personal participant information like IP address, software system, or region. Completing the questionnaire took 10 to 15 min.

The survey contained 34 questions (data from 18 of these questions will be reported in the current manuscript^2^) and comprised three parts. Part one focused on the participants’ demographics (e.g., age, sex, educational level, personal migration background) and relevant job information (e.g., profession, experience). Part two asked participants about their clinical experience with refugees and asylum seekers as well as with other patients in the past 12 months (e.g., how many patients were treated by the participant, how long the average patient admission waiting time was, how many patients dropped out of treatment due to financial reasons, etc.). The third part was only activated if participants reported they had treated at least one refugee or asylum seeker in the past 12 months. This (third) part covered questions regarding the treatment of refugees or asylum seekers (e.g., how many refugees or asylum seekers had been treated by the participant, how often the therapist used interpreter services for the treatment of refugees and asylum seekers, participants’ experiences with interpreters, previous or current work experience at an institution focused on the psychotherapeutic treatment of refugees and asylum seekers, and the circumstances that prevented the participants from treating this target population).

Some of the questions were closed, whereas others, for example, the question regarding barriers to treating refugees and asylum seekers (“Are there circumstances that prevent you from more frequent psychiatric/psychotherapeutic treatment of refugees?”), were open-ended to prevent biasing the participants’ responses. Items that were analyzed for the present manuscript are presented in Table 1.^3^

**Table 1:** Items in the survey

Nr.	Question	Answer type	Scaling
1.	Would you like to take part in the survey or just have a look at the questions?	sc	n
2.	What is your (main) occupation?	sc	n
3.	Where do you primarily work? i.e. where do you spend the majority of your professional life?	sc	n
4.	Please state your workload related to your psychiatric-psychotherapeutic activities. 100% = full-time, 5 days a week	ni	i
5..	How many clients (psychiatric/psychotherapeutic) do you treat per year on average as a part of your main occupation? Please estimate the number of clients [In absolute terms, NOT adjusted to a 100% workload]	ni	i
6.	How many sessions of psychotherapy have you conducted on average per person? (past 12 months) If not applicable, please enter 0	ni	i
7.	Please estimate the average waiting time for a person to commence therapy in your care (past 12 months) in weeks / if none, please enter 0	ni	i
8.	Please estimate how many persons you had to reject due to capacity reasons (past 12 months) in percent % of new registrations / if none, please enter 0	ni	i
9.	Please estimate the number of persons that you have treated who have gone through an asylum procedure or are currently in one (past 12 months) if none, please enter 0	ni	i
10.	Please think about your clients: What percentage, measured by the following sub-groups, has made use of any translation aids during treatment? (past 12 months)	sc	i
11.	Was the translation aid usually sufficient for optimal communication with the client? (past 12 months)	sc	r
12.	How often (in percent) were the following translation aids used in situations that required translation? (past 12 months)	ni	i
13.	Are you, or have you ever, worked at an institution focused on psychotherapeutic treatment of refugees? e.g.: an outpatient clinic for victims of torture or war; consultation hours for migrants, etc.	sc	n
14.	Are there circumstances that prevent you from more frequent psychiatric/psychotherapeutic treatment of refugees or asylum seekers?	oe	s
15.	How old are you?	ni	i
16.	Gender	sc	n
17.	Since when have you been working full-time in your current profession?	ni	n
18	Please enter the postal code of your main place of work	ni	n

*mc* multiple choice, *sc* single choice, *ni* numeric input, *oe* open-end, *i* intervalscaled, *o* ordinal scaled, *n* nominal scaled, *s* string

### Definition and calculation of variables for statistical analyses

The invitation to participate in the survey was spread not only among psychiatrists and psychotherapists but also among all Swiss psychiatric hospitals and institutional providers of mental health care. For this reason, it was possible that participants working in an in-patient or non-medical service setting or participants from other professional groups (e.g., nurses, social workers, etc.) participated in the survey. Therefore, these participants and participants with zero patients in the last 12 months were excluded from the final data analyses.

#### Aim 1 (exploring the proportion of providers treating asylum seekers and refugees)

A proportion coefficient (indicating the ratio between the number of refugees or asylum seekers and the total number of patients treated during the past 12 months) was calculated. In addition, we defined three subgroups: the subgroup (out of the total number of providers) of frequent treaters (participants who indicated they had treated at least 10 (≥ 90th percentile of the number of treated refugees or asylum seekers in the total sample) refugees or asylum seekers during the past 12 months), the subgroup including providers who did not treat a single patient from the target population (no treater group), and the third subgroup of providers who had treated between one and nine refugees or asylum seekers in the past 12 months (treater group). Furthermore, we defined a specialized subgroup (out of the frequent-treater group) of providers who, in addition to having treated at least 10 refugees or asylum seekers during the past 12 months, reported that they are working currently or have previously worked in a center that offers psychotherapeutic treatment of refugees or asylum seekers.

#### Aim 2 (circumstances under which treatment is provided)

Participants had indicated the frequency (%) (constant sum question) they had used different translation options (family member, friend, someone from a refugee organization, trained interpreter, software) during the past 12 months. The translation options referring to untrained translation options (family member, friend, someone from a refugee organization, software) were summed up under the category “untrained interpreters.” Satisfaction with translational services was assessed by three variables [ranging from 1 (not enough) to 2 (comprehension ensured), and 3 (good translation)] related to i) the understanding of patients’ concerns, ii) the communication with patients, iii) the provider’s ability to provide treatment-relevant information to the patient. Based on these three variables (i, ii, iii), the average (across i, ii, and iii) satisfaction (iv) was calculated.

#### Aim 3 (barriers to treating more refugees and asylum seekers)

Answers to the open-ended question (Table 1, item 14) regarding the circumstances that prevent participants from providing more frequent treatment to refugees (aim 3 of the study) were analyzed according to the standard procedures [48, 49] as follows: First, a master’s student created the codes and integrated their grouping into the broader concept. Next, the research team reviewed and accepted the coding framework. Finally, a Ph.D. student (first author) not involved in the first step coded the data set according to this coding framework.

### Statistical analysis

All descriptive analyses were performed with SPSS 25 [50]. For the analysis of open-ended questions, we additionally used QDA Miner Lite [51] and Microsoft EXCEL. None of the assessed variables were normally distributed. We will, therefore, report median (*Mdn)* and quartiles (Q_1_, Q2, Q_3_, Q_4_).

#### Aim 1

Next to exploring the descriptive numbers of the total sample and the four defined subgroups (frequent treaters, treaters, no treaters, specialized subgroup) of refugees and asylum seekers that participants had treated in the past 12 months, the group sizes of the four subgroups were calculated. In addition, the above-described proportion coefficient was used to assess how frequently psychiatrists and psychotherapists had treated the target population.

#### Aim 2

Spearman’s correlations were calculated to analyze the relationship between the number of refugees and asylum seekers treated and the proportion coefficient for the past 12 months with (2a) treatment waiting time (in weeks) and (2b) the percentage of patients rejected for capacity reasons during the past 12 months. Furthermore, in addition to exploring the frequency of use of translation services and providers’ general satisfaction with translation services, Spearman’s correlations were used to analyze the relationship between the frequency with which participants used trained (and untrained) interpreters and participants’ satisfaction with translation services (2c) in the past 12 months. To analyze whether participants predominantly working with trained translators (≥ Q_3_) differ from participants mostly working with untrained translators (< Q_3_) in their satisfaction with translational services, we used the Mann-Whitney-U-Test (2d).

#### Aim 3

Besides the descriptive analysis of the reported circumstances, the following exploratory analyses were performed to better understand the assessed circumstances preventing participants from more frequently treating refugees or asylum seekers (question 14, Table 1): Mann-Whitney U-Tests were used to examine the relationship (i) between the frequency of treatment of refugees and asylum seekers (reported numbers as well as proportion coefficient) and the explicit denial of the existence of any barriers (no barriers listed in response to question 14) and (ii) between the professional subgroups (psychiatrists vs. psychotherapists) and the number of reported barriers. Spearman’s correlation was used to calculate the correlation between the number of cited barriers and the number of treated refugees or asylum seekers (reported numbers as well as proportion coefficient). Finally, the relationship between the citing of a particular barrier and the professional subgroup, as well as the employment setting (self-employed versus employed), was analyzed by means of Chi-squared tests.

## Results

### Sample characteristics

**Fig. 1:**
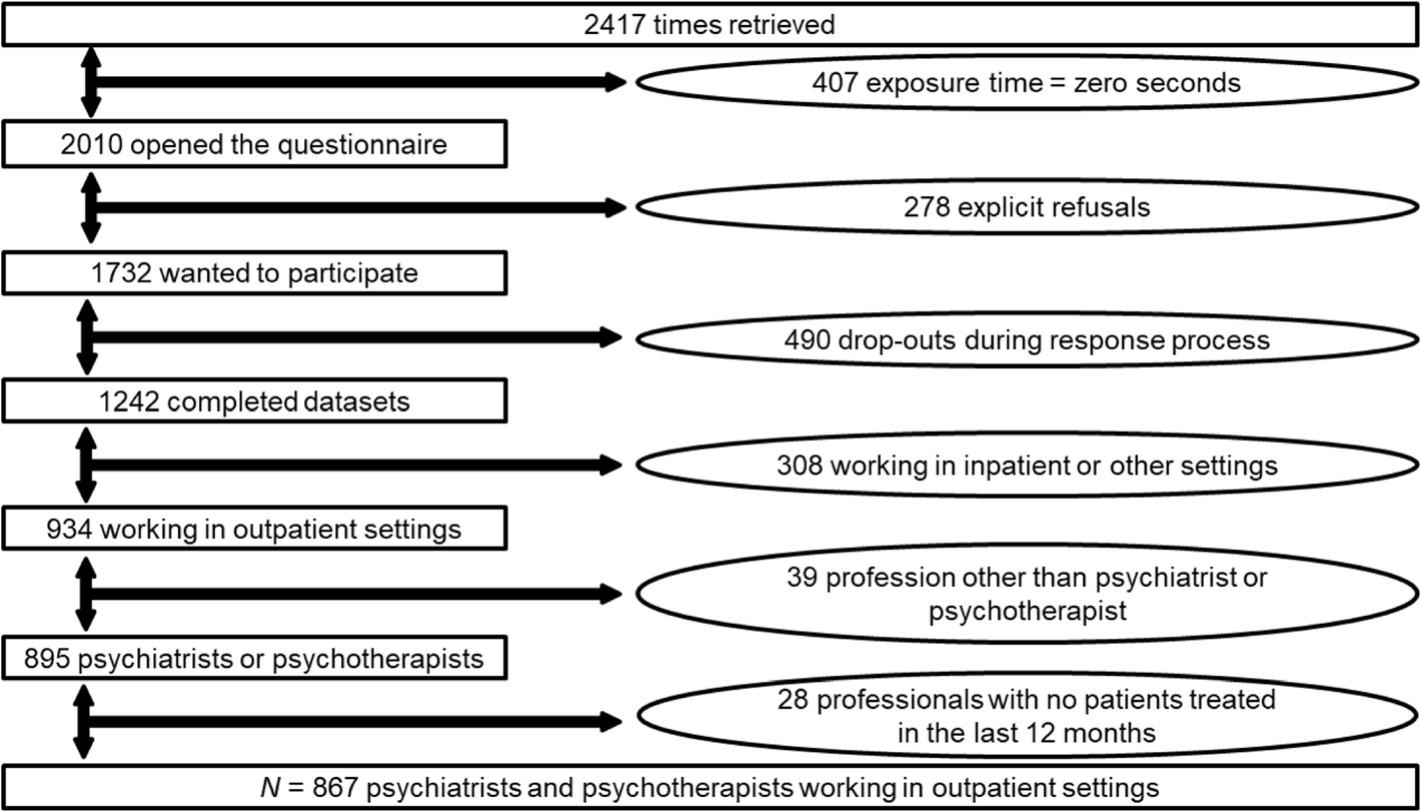
Participant flowchart

The questionnaire was accessed 2417 times in total. A total of *n* = 1242 providers (including providers from in-patient or other settings) completed the survey. However, for the present analyses, only providers working in an outpatient setting (including self-employed providers outside of institutions) were included. Participants working in in-patient settings or non-medical service settings and participants with zero patients in the last 12 months were excluded. Due to the reasons detailed in Fig. 1, the final sample size for the present analyses resulted in *N* = 867 cases.^4^ The response rate for this final sample was 13.5%, based on the number of individuals who received a personal email invitation. This corresponds to 7.9% of the official total number of psychiatrists (4430) and psychotherapist (6542) working in Switzerland [52, 53].

Five hundred and eighty-six participants (67.6%) were female. The median age was 49 (Q_1_: 39.0, Q_3:_ 58.0), and the median professional experience was 16 years (Q_1_: 9.0, Q_3:_ 25.0). The distribution of providers (psychotherapists vs. psychiatrists) in our sample did not differ from the general population of psychotherapists and psychiatrists (χ^2^(1, *N* = 867) = 1.394, *p* = .238).^5^ Although significantly younger (*z* = − 15.507, *p* < .001), our sample was comparable regarding gender distribution with the general population of Swiss psychiatrists working in outpatient settings.^6^

Five hundred and thirty-four respondents (61.6%) were psychotherapists or psychotherapists in training. Mostly, respondents were self-employed working in the field of psychiatry (*n* = 372, 42.9%) or an outpatient psychiatric facility (*n* = 275, 31.7%). The median level of employment was 79% (Q_1_: 50.0, Q_3:_ 90.0). In the past 12 months, participants had treated a median of 50 patients^7^ (Q_1_: 30.0, Q_3:_ 80.0), with a median of 20 sessions^8^ (Q_1_: 14.0, Q_3:_ 30.0) per patient. Based on the analysis of collected postal codes, 79.6% of the respondents were from the German-speaking part of Switzerland, 18.3% from the French-speaking part, and 3.2% from the Italian-speaking part. Table 2 provides further participant characteristics.

**Table 2:** Job-related characteristics of participants

Variable	Number	Percent
Female psychiatrists	160	48.2
Female psychotherapists	426	80.1
Profession		
Psychiatrists	281	32.4
Psychiatric residents	52	6.0
Psychotherapists	468	54.0
Psychotherapists in training	66	7.6
Place of work		
Self-employed	417	48.1
Employed	439	50.6
Other	11	1.3

The table presents the number of participants from the subgroups (incl. % of the total sample, *N* = 867)

### Treatment frequency (aim 1)

The median number of refugees or asylum seekers treated in the past 12 months was 1 (Q_1_: .0; Q3: 4.0) in the total sample. As shown in Table 3, less than half of the participants had not treated any refugees or asylum seekers during the past year. A percentage of 12.7% was identified as representing the frequent-treater subgroup (treatment of 10 or more refugees or asylum seekers during the past 12 months) of psychiatrists and psychotherapists. Forty-two persons (38.5%) from the latter group indicated that they had experience working in a center specialized in the treatment of refugees and asylum seekers (specialized subgroup). When basing our analyses on the proportion coefficient, we found that, overall, a median of 1% (Q_1_: 0.0; Q_3:_ 5.0%) of the total sample’s patients were refugees or asylum seekers. Median proportion coefficients (Q_1_; Q_3_) for the no-treater group, for the treater group, and for the frequent-treater group were 0.0 (0.0; 0.0), 4.0% (2.5%; 7.14%), and 25.0% (Q_1_: 14.29%; Q_3:_ 46.0%) (specialized subgroup: 43.0% (20.0%; 90.0%); non-specialized subgroup: 20.0% (13.3(3)%; 31.67%)).

**Table 3:** Number (percentages) of mental health providers depending on frequency of treatment of refugees and asylum seekers

	total sample	no treatment (0 pat./year)	treatment (1–9 pat./year)	frequent treatment (≥ 10 pat./year)	
				non-specialized^a^	specialized^b^
Psychotherapists	533 (61.6%)	270 [69.8%] < 50.7%>	215 [58.3%] < 40.3%>	27 [40.3%] < 5.1%>	21 [50%] < 3.9%>
Psychiatrists	332 (38.4%)	117 [30.2%] < 35.3%>	154 [41.7%] < 46.4%>	40 [59.7%] < 12.0%>	21 [50%] < 6.3%>
Total Sample	865 (100%)	387 (44.7%)	369 (42.7%)	67 (7.7%)	42 (4.9%)

2 participants (pat.) are not included due to missing values; ^a^ providers without work experience in specialized center; ^b^ providers with experience in specialized center; (%) – related to the total sample; <% > − related to the subsample of providers (psychotherapists or psychiatrists); [%] - related to the subsample (providers with no treated refugees or asylum seekers in the past 12 months, providers with 1–9 treated refugees or asylum seekers in the past 12 months, specialized and non-specialized providers with 10 and more treated refugees or asylum seekers in the past 12 months)

### Waiting times and rejections for capacity reasons (aims 2a and 2b)

The median waiting times for an admission of a new patient and the percentage of rejections for capacity reasons during the past 12 months are shown in Table 4. The correlation between the number of refugees and asylum seekers and waiting times was significant for both the total number of treated refugees or asylum seekers (*r*_*s*_ = .164, *p* < .001) and the proportion coefficient (*r*_*s*_ = .175, *p* < .001).

**Table 4:** Median waiting times for the admission of a new patient and percentage of rejections for capacity reasons depending on the assessed subgroup

	Waiting time (weeks) [Q_1_;Q_3_]	% rejection due to capacity [Q_1_;Q_3_]
total sample	3 [1;5]	5 [0;20]
no treatment (0 pat./year)	2 [1;4]	5 [0; 20]
treatment (< 10 pat./year)	3 [2;6]	5 [0;20]
frequent treatment (≥ 10 pat./year)	3 [2; 7]	5 [0; 30]
non-specialized^a^	3 [2; 8]	2 [0; 20]
specialized^b^	3 [2; 4.25]	20 [0; 35]

^a^ providers without work experience in specialized center; ^b^ providers with experience in specialized center

The percentage of rejections was not significantly correlated with the total number of refugees or asylum seekers treated or the proportion coefficient (all *p*’s > .764). However, Table 4 (*n* = 42) suggests that there was a higher median percentage of rejections in the specialized (n = 42) compared to the non-specialized (*n* = 67) subgroup. We, therefore, conducted an additional, unplanned Mann-Whitney-U-Test test, showing that this group’s difference was statistically significant (z = − 2.434, *p* = .015).

### Use of and satisfaction with interpreters (aims 2c and 2d)

Participants working with refugees or asylum seekers used interpreter services for the treatment of almost every other refugee or asylum seeker (*Mdn* = 40.0 [frequency of interpreter services in %]). With *Mdn* = 80.0 (Q_1_: 60.0; Q_3:_ 90.0), this rate was significantly higher for providers from the specialized subgroup (z = − 4.302, *p* < .001). Participants indicated that they used trained interpreters at a median rate of 80% (Q_1_: 40.0; Q_3:_ 100) and untrained interpreters at a median rate of 10% (Q_1_: .0; Q_3:_ 50.0) to treat refugees or asylum seekers. As shown in Table 5, satisfaction regarding all assessed aspects of translation was highly positively correlated with the frequency of utilization of trained interpreters and highly negatively correlated with the frequency of utilization of untrained interpreters. Average satisfaction was significantly higher for providers predominantly (≥ Q_3_) using trained interpreters than for providers using trained interpreters less frequently (< Q_3_) (z = − 3.215, *p* = .001). In parallel, providers predominately (≥ Q_3_) using untrained interpreters showed significantly lower average levels of satisfaction with translations (z = − 5.351, *p* < .001) than providers using untrained interpreters less frequently (< Q_3_). The latter two findings remained unchanged when looking at the separately assessed aspects of satisfaction with translations (all *p’*s < .028).

**Table 5:** Relationship (Spearman correlation coefficients) between the frequency of use of translation options and provider’s satisfaction with different translation aspects

	understanding concerns	communication with patients	providing information	averaged satisfaction
trained interpreter	.356*	.316*	.274*	.351*
	(*n* = 295)	(*n* = 296)	(*n* = 288)	(n = 296)
untrained interpreter	−.266*	−.241*	−.210*	−.271*
	(n = 295)	(n = 296)	(n = 288)	(n = 296)

* *p* < .001

### Barriers to more frequent treatment of refugees and asylum seekers (aim 3)

Four hundred and ten of the total 867 participants (47.3%) answered the question regarding the circumstances preventing them from treating refugees or asylum seekers more frequently (Table 1, item 14). This question was not mandatory; i.e., it was possible to skip this question to prevent dropouts.

Overall, the most frequently mentioned barriers were “lack of funding for treatment” (*n* = 102 (24.9%^9^)), “lack of funding for interpreters” (*n* = 88 (21.5%^9^)), and language-related barriers (*n* = 62 (15.1%^9^)). Table 6 shows the aggregated codes of the answers. As indicated by Table 6, roughly 60% of those answering this question were psychotherapists. Participants who had answered this question described between one and four barriers to more frequent treatment (*Mdn* = 1.0). Fifty-six respondents (13.7%) indicated that no barriers were preventing them from treating refugees or asylum seekers more frequently. The frequency of responses indicating there were “no barriers” was unrelated to the reported number of patients and the proportion coefficient for the target population (z = −.376, *p* = .707; z = −.957, *p* = .338). Psychiatrists cited significantly more barriers to more frequent treatment than psychologists (*z* = − 3.258, *p* = .001). Correlations between the number of cited barriers to more frequent treatment and the frequency of treatment of refugees and asylum seekers (reported numbers as well as proportion coefficient) were neither found for psychotherapists nor psychiatrists. However, among the participants who answered this question, there were significant correlations between the frequency of treatment of refugees and asylum seekers (reported number as well as proportion coefficient) and the number of barriers to more frequent treatment that were noted (*r*_s_ = .113, *p* = .033, *n* = 356 resp. *r*_s_ = .129, *p* = .019, *n* = 331).

**Table 6:** Circumstances preventing psychiatrists and psychotherapists from more frequent psychiatric/psychotherapeutic treatment of refugees or asylum seekers

Barriers (based on coding framework)	Total (*n* = 410)	Psychotherapists (*n* = 244)	Psychiatrists (*n* = 166)
	n (%)	n (%)	n (%)
Lack of funding for treatment	102 (24.9%)	84 (20.5%) [82.4%]	18 (4.4%) [17.6%]
Lack of funding for interpreters	88 (21.5%)	57 (13.9%) [64.8%]	31 (7.6%) [35.2%]
Language	62 (15.1%)	36 (8.8%) [58.1%]	26 (6.3%) [41.9%]
No contact with the target population	47 (11.5%)	23 (5.6%) [48.9%]	24 (5.9%) [51.1%]
Capacity	43 (10.5%)	25 (6.1%) [58.1%]	18 (4.4%) [41.9%]
Expenditure of time for administration	33 (8%)	11 (2.7%) [33.3%]	22 (5.6%) [66.6%]
No own experience/qualification	21 (5.1%)	14 (3.4%) [66.6%]	7 (1.7%) [33.3%]
Therapist’s emotional distress too high	19 (4.6%)	13 (3.2%) [68.4%]	6 (1.5%) [31.6%]
Availability of interpreters	19 (4.6%)	6 (1.5%) [31.6%]	13 (3.2%) [68.4%]
Insecure residency status	14 (3.4%)	6 (1.5%) [42.9%]	8 (2.0%) [57.1%]
Cultural barriers	12 (2.9%)	6 (1.5%) [50%]	6 (1.5%) [50%]
Complexity of treatment	12 (2.9%)	5 (1.2%) [41.6%]	7 (1.7%) [58.3%]
Social problems of the patients	11 (2.7%)	5 (1.2%) [45.5%]	6 (1.5%) [54.5%]
Frequent relocation	6 (1.5%)	2 (< 1%) [33.3%]	4 (< 1%) [66.6%]
Lack of motivation (of providers)	4 (< 1%)	1 (< 1%) [25%]	3 (< 1%) [75%]
Other	34 (8.3%)	12 (2.9%) [35.3%]	22 (5.4%) [64.7%]
Sum	526	305	221

(%) – related to n = 410; [%] – related to the n for the barrier; Values rounded of/up

Results of subgroup analyses showed that a lack of funding was more frequently reported by psychotherapists (84 out of 102) than by psychiatrists (χ^2^(1, *n* = 867) = 21.063, *p* < .001). Self-employed psychotherapists (*n* = 236), furthermore, reported lack of funding significantly more often (62 out of 84 times) than employed psychotherapists (*n* = 289) (χ^2^(1, *n* = 525) = 35.253, *p* < .001). In contrast, self-employed psychiatrists (*n* = 181) reported capacity problems significantly more often (14 out of 18 times) than employed psychiatrists (*n* = 151) (χ^2^(1, *n* = 332) = 4.153, *p* = .042). Psychiatrists reported availability of interpreters (13 of 166 vs. 6 of 244) and expenditure of administration time as barriers (22 of 166 vs. 11 of 244) more frequently than psychotherapists (χ^2^(1, *N* = 867) = 7.397, *p* = .007 resp. χ^2^(1, *N* = 867) = 11.580, *p* = .001).

## Discussion

To our knowledge, this is the first attempt to gain insights into outpatient mental health care for refugees and asylum seekers in Switzerland based on reports from psychiatrists and psychotherapists working in an outpatient mental health care setting. Our findings show there was a low number of rejections of patients for capacity reasons and short median waiting times for the admission of new patients. They thus indicate that outpatient primary mental health care providers may have the capacity to treat more refugees and asylum seekers and unburden specialized treatment centers. However, barriers such as lack of funding of interpreters are a hindrance.

The perspectives of psychiatrists and psychotherapists working in an outpatient mental health care setting have yet to be investigated or considered in discussions related to mental health care provision to refugees and asylum seekers in Switzerland. Previous studies were based primarily on data obtained from refugees and asylum seekers and did not focus on the providers of mental health care for refugees and asylum seekers in Switzerland [6, 13, 14, 27, 29, 30, 36, 41, 54–57]. Only six studies assessed the opinions of health or social workers or caregivers [4, 34, 35, 40, 42, 58]. Only one survey and two qualitative reports recruited medical employees from the transcultural sector of mental health care [4, 35, 40]. However, this survey was conducted only in the French part of Switzerland, and almost half of the sample included nurses who do not provide mental health treatment. Less than 20% of the sample was represented by psychiatrists or psychotherapists, and samples of both qualitative reports were very small.

Despite our rather large sample and satisfactory (but somewhat low) response rate for an email survey [59], a major limitation of the current study concerned the representativeness of the sample. Nevertheless, the distribution of providers (psychotherapists vs. psychiatrists) in our sample did not differ from that of the general population of psychotherapists and psychiatrists, and the gender distribution of psychiatrists was comparable to the official data. However, psychiatrists participating in the survey were significantly younger. It is, furthermore, possible that our survey appealed more to providers working with refugees and asylum seekers, and they were thus overrepresented in our sample. Our explicit invitation of providers who are inexperienced in working with refugees and asylum seekers may have counteracted this tendency. In fact, the proportion of refugees or asylum seekers relative to the total number of patients did not exceed 10% for the large majority (92%) of our participants. At the same time, the representativeness of the sample is not of major importance when assessing barriers that hinder providers from treating more refugees and asylum seekers. For the assessment of barriers, it is, in fact, beneficial to have both sufficient numbers of providers who are non-treaters (but show a certain interest in the topic, as reflected by their participation) and providers who are experienced with treating refugees and asylum seekers.

However, in addition to the above-mentioned aspects of representativeness, another limitation is the reduced number of responses to questions regarding the circumstances that prevent more frequent treatment of refugees or asylum seekers (question 14, Table 1), which was optional and, unexpectedly, was skipped by a large proportion of our sample. Our survey included three questions related to the issue of translation. These questions were asked prior to the open-ended question on potential barriers. These precursory questions might have influenced the participants’ answers to the open question by encouraging them to list translation-related issues as a significant barrier. Furthermore, we did not differentiate between refugees and asylum seekers. It thus cannot be ruled out that participants’ answers were affected by the population that respondents had in mind at the time of completing the survey. Finally, some of the questions required participants to estimate certain numbers, e.g., regarding average waiting times and rejection quotes. For some participants, for example, those not scheduling their appointments themselves, it may have been difficult to make an estimation, limiting the reliability of our findings.

### Treatment frequency, waiting times, and rejections for capacity reasons

Despite these limitations, our study allows for the drawing of tentative conclusions on the circumstances (e.g., length of waiting lists, use of and satisfaction with interpreter services) under which psychiatrists and psychotherapists provide outpatient treatment to refugees or asylum seekers in Switzerland. More specifically, our findings detail the barriers that prevent providers from treating more refugees and asylum seekers. The majority of providers (55.3%) reported having treated refugees or asylum seekers in the past 12 months. Moreover, almost a quarter of them had treated at least 10 individuals with a refugee or asylum-seeker background during the past 12 months. Surprisingly, only 13 participants (1.5%) reported waiting times longer than six months, and the median waiting time for admission to treatment was three weeks across all subsamples. While the information on waiting times in general is lacking for psychiatrists, waiting times in our sample were well in line with a survey of Swiss psychotherapists conducted in 2012 [60]. Despite the rather short waiting times, we found significant correlations between the waiting time and the number of treated refugees and asylum seekers for both proportion coefficients and the reported total number of treated refugees and asylum seekers. However, the corresponding effect sizes were extremely small (*r*’s < .03), limiting the practical relevance of this finding. The frequency of rejections for capacity reasons in our sample is comparable to the rate of rejections reported by psychotherapists [60], but, again, comparative data for psychiatrists is unavailable. While the rate of rejections was not correlated with the number or proportion of treated refugees or asylum seekers, the specialized subgroup reported a significantly higher percentage of rejection (median: 20%) than the non-specialized subgroup of providers. This indicates that providers specialized in the treatment of refugees and asylum seekers may report short waiting times because patients are rejected for capacity reasons rather than put on a waiting list, knowing that patients could stay on the waiting list for years with no guarantee of treatment [41, 61]. In fact, we found a moderate positive relationship between waiting times and percentages of rejection for providers specialized in treating refugees and asylum seekers (*r*_*s*_ = .523, *p* < .001).

Forty-five percent (44.7%) of our participants did not treat a single refugee or asylum seeker in the past 12 months. This subgroup reported the lowest waiting time (*Mdn* = 2.0 weeks) and a low rejection rate (*Mdn* = 5.0%). These results suggest that psychiatrists and psychotherapists working in outpatient settings might have the capacity to treat more of those asylum seekers and refugees who are rejected by specialized treatment units. However, more research is needed to further explore the potential of such an approach. For example, future studies should examine whether there are additional barriers that arise as part of the treatment process and may not have been mentioned by the providers completing our survey.

### Use of and satisfaction with interpreters

Our findings, furthermore, show that for the treatment of refugees and asylum seekers, the assessed providers seemed to depend on interpreters as they used them for almost every other patient with a refugee or asylum seeker background. The fact that specialized providers used interpreters in four out of five cases illustrates this dependency even more clearly. Mostly, participants used professionally trained interpreters and were satisfied with the quality of the translation. At the same time, for those often working with lay-interpreters, the quality of translations was non-satisfactory. To enable communication – that is crucial for providing of adequate treatment [43, 46] – the financing of professional interpreters thus needs to be covered. Efforts to implement public funding are currently underway in some cantons, but it still much too early to speak about nationwide coverage. Indeed, the results on the usage of trained interpreters show how important trained interpreters are to the provision of appropriate mental health care for refugees and asylum seekers in our country.

### Barriers to more frequent treatment of refugees and asylum seekers

Unexpectedly, lack of funding for treatment was the most frequently mentioned (by 24.9%) barrier. Nevertheless, in line with our prediction regarding perceived barriers that prevent providers from treating refugees and asylum seekers more frequently, language-related obstacles were often cited (lack of funding for interpreters by 21.5% of participants and language by 15.1% of the participants answering the question regarding barriers). This is in line with research showing that even after years of being in Switzerland, the majority of refugees or asylum seekers and doctors are not able to sufficiently communicate with each other [39, 41]. Our results on participants’ satisfaction with untrained interpreters as well as studies demonstrating various problems including misunderstandings, stigma, imprecise answers, and medical complications (due to loss of crucial information or incorrect translations that make time-sensitive or correct diagnosis impossible) [43, 44] make it clear that the use of untrained, unpaid interpreters is an inadequate alternative to the use of trained interpreters.

Lack of funding for treatment was most frequently cited by psychotherapists. This finding might be related to the fact that in Switzerland, psychotherapy carried out by a psychologist is currently not covered by mandatory basic medical insurance if not delegated by a physician working within the same private practice or institution. This limits access to mental health care, in particular for patients with limited financial resources. In line with this interpretation, this barrier was most frequently mentioned by self-employed psychotherapists.

Observations noting capacity restraints as well as lack of time for administration for more frequent treatment of this target population were relatively uncommon. These barriers were mentioned by only 10.5 and 8% of participants, respectively. This fact underlines our assumption that psychiatrists and psychotherapists working in outpatient settings would have the capacity to treat more of those asylum seekers and refugees who have been rejected by specialized treatment units.

Interestingly, asylum seeker- and refugee-specific issues (e.g., frequent relocation, insecure residency status, transcultural differences, social problems, the complexity of (trauma) disorder) were mentioned only a few times. However, possibly, some of these issues were overlooked due to the perception of the target population respondents held at the time of completing the survey. Finally, it is necessary to keep in mind that the current study assessed barriers (circumstances) that prevent providers from more frequently treating refugees and asylum seekers (before the treatment) but not barriers arising while patients were already in treatment [62–64].

Over and above structural barriers to treatment such as availability and funding of qualified interpreters, or insurance coverage of psychotherapists, socio-cultural barriers are to be considered when treatment is enabled. Several studies showed that the cross-cultural setting might be a challenge to provide appropriate mental health care to refugees and asylum seekers for a variety of reasons such as stigma, taboo, trust issues, and a mismatch between Western concepts of diagnosis and treatment and the problems and needs perceived by refugees and asylum seekers [4, 40, 65–72]. Moreover, the expression of the perceived problems, idioms of distress, or symptom expression of common mental disorders can vary substantially within and between cultural backgrounds and may decrease the accuracy of diagnostic appraisals and treatment outcome [73–76]. Therefore, a culturally sensitive treatment approach considering i.a. the wide variety of models of disease, idioms of distress, interactional habits and role models of patients and therapists is indispensable [77, 78].

While structural and socio-cultural barriers need to be addressed in order to facilitate appropriate and timely treatment for refugees and asylum seekers, not every refugee with mental health problems requires specific (and therefore usually more expensive) care in facilities specialized on trauma or transcultural psychotherapy. Rather, these centres could focus their limited capacity primarily on complex and severe cases if regular mental health care institutions and psychiatrists and psychotherapists in private practice could be enabled to take over suitable cases by virtue of appropriate training and supervision. Besides capacity reasons, such a shift into regular treatment structures might contribute to a favourable familiarization of health care systems and hosting societies with regard to refugee issues.

## Conclusions

Notwithstanding the above-mentioned limitations, the study provides a first and unique insight into outpatient mental health care for refugees and asylum seekers in Switzerland based on survey data from psychotherapists and psychiatrists. According to our data, it seems unlikely that extended waiting times and rejections for capacity reasons are the main factors affecting access of refugees and asylum seekers to mental health care. Instead, a lack of interpreter services seems to be a considerable barrier preventing mental health care providers from treating more refugees and asylum seekers. On the one hand, psychotherapists and psychiatrists are highly dependent on qualified interpreters. On the other hand, financial coverage is unregulated (except for some cantons), which may have contributed to the low numbers of patients with a refugee background reported to be treated by our study participants. Similar findings have been emphasized in other Western European countries [62, 79, 80]. As suggested by our results, another opportunity for improving access of refugees and asylum seekers to mental health care in Switzerland might be to introduce mandatory health insurance coverage of psychotherapy by self-employed psychotherapists, as proposed by the *Anordnungsmodell*, which is a focus of ongoing political initiatives and debates. From a long-term perspective, the inclusion of training in transcultural competence and culture-sensitive treatment in the education of psychiatrists and psychotherapists might help to overcome the treatment gap in the future. Investing in the improvement of vulnerable populations’ access to mental health care is essential not only from an ethical viewpoint. Effectively addressing mental health problems is a significant contributor to health and well-being at both an individual and social level, therefore ensuring the future economic strength and stability of our society [41].

## Supplementary information

**Additional file 1.** Questionnaire of the survey.

## Data Availability

The datasets and the original questions in German, French, and Italian are available upon reasonable request from the corresponding author.

## Abbreviations

PTSD: Posttraumatic Stress Disorder
FOPH: Federal Office of Public Health
SEM: State Secretariat for Migration
FMH: Swiss Medical Association
WBV: Weiterbildungsverein Psychiatrie und Psychotherapie Zürich, Zentral-, Nord- und Ostschweiz [Swiss organization providing post-graduate education and training for residents specializing in psychiatry]
FSP: Federation of Swiss Psychologists
SBAP: Swiss Society for Applied Psychology
ASP: Association of Swiss Psychotherapists
BASEC: Business Administration System for Ethics Committees [in Switzerland]
PsyReg: Register of psychotherapists [official online data bank of psychotherapists in Switzerland]

## Footnotes

1 NPO treats severely traumatized refugee children in Amman, Jordan.

2 Several questions about, e.g., the working language of providers or origin of the patients with refugee backgrounds as well as regarding frequently diagnosed disorders were not analyzed as part of the present manuscript but used for setting up a different research project.

3 The corresponding author will provide the original questions in German, French, and Italian upon request.

4 The dataset supporting the conclusions of this article is included in the article (and its additional file).

5 Based on information from the official register of psychotherapists (PsyReg) and the register of the FMH [52, 53].

6 Based on information regarding age and gender that is only available for psychiatrists (but not psychotherapists) working in outpatient settings [52].

7 89 answers were identified as outliers (Q_3_ + 1.5*IQR) and excluded from this analysis due to illogical values.

8 145 answers were identified as outliers (Q_3_ + 1.5*IQR) and excluded from this analysis due to illogical values.

9 Percentages in the reported result are based on the 410 participants who answered the question regarding treatment barriers.
