# Supplementary material for: Barriers to access to outpatient mental health care for refugees and asylum seekers in Switzerland: the therapist’s view
Source: BMC Psychiatry. 2020 Jul 17;20:378. doi: 10.1186/s12888-020-02783-x (PMC7366894; doi:10.1186/s12888-020-02783-x)
Supplement: Supplementary file 1 — Additional file 1. Questionnaire of the survey. [file 12888_2020_2783_MOESM1_ESM.docx]

*Questionnaire of the survey*

| Nr. | Question | Answer type | Single-choice answer possibilities | Scaling | |
| --- | --- | --- | --- | --- | --- |
|  | Would you like to take part in the survey or just have a look at the questions? | sc | I want to participate  I just want to have a look at the questions  I do not want to participate (I have experiences in treating refugees or asylum seekers)  I do not want to participate (I do not have any experiences in treating refugees or asylum seekers) | n |  |
|  | What is your (main) occupation? | sc | Psychiatrist  Psychiatrist resident  Other physician  Other physician resident  Psychotherapist  Psychotherapist in training  Psychologist  Other | n |  |
|  | Where do you primarily work?  i.e. where do you spend the majority of your professional life? | sc | In-patient hospital or clinic (psychiatric department)  Outpatient hospital or clinic (psychiatric department)  In-patient hospital or clinic (other department)  Outpatient hospital or clinic (other department)  Self-employed in practice (psychiatry)  Self-employed in practice (other field)  Employed in practice (psychiatry)  Employed in practice (other field)  Other | n |  |
|  | Please state your workload related to your psychiatric-psychotherapeutic activities.  100 % = full-time, 5 days a week | ni | - | i |  |
|  | How many clients (psychiatric/psychotherapeutic) do you treat per year on average as a part of your main occupation?  Please estimate the number of clients [In absolute terms, NOT adjusted to a 100% workload] | ni | - | i |  |
|  | How many sessions of psychotherapy have you conducted on average per person? (past 12 months)  If not applicable, please enter 0 | ni | - | i |  |
|  | Please estimate the average waiting time for a person to commence therapy in your care (past 12 months)  in weeks / if none, please enter 0 | ni | - | i |  |
|  | Please estimate how many persons you had to reject due to capacity reasons (past 12 months)  in percent % of new registrations / if none, please enter 0 | ni | - | i |  |
|  | Please estimate the number of persons that you have treated who have gone through an asylum procedure or are currently in one (past 12 months)  if none, please enter 0 | ni | - | i |  |
|  | Please think about your clients:  What percentage, measured by the following sub-groups, has made use of any translation aids during treatment? (past 12 months)  Sub-groups:   - persons with migration background with no experience as refugee or asylum seeker - persons with migration background with experience as refugee or asylum seeker), | sc | 10%  20%  30%  40%  50%  60%  70%  80%  90%  100% | i |  |
|  | Was the translation aid usually sufficient for optimal communication with the client? (past 12 months)  Regarding to:   - the understanding of patients' concerns - the communication with patients - the provider's ability to provide treatment-relevant information to the patient | sc | For each sub-item:  Not enough  Comprehension ensured  Good translation | r |  |
|  | How often (in percent) were the following translation aids used in situations that required translation? (past 12 months)  Options:   - Family member - Friend - Someone from a refugee organization - Trained interpreter - Software - Other | ni | constant sum question | i |  |
|  | Are you, or have you ever, worked at an institution focused on psychotherapeutic treatment of refugees?  e.g.: an outpatient clinic for victims of torture or war; consultation hours for migrants, etc. | sc | Yes  No | n |  |
|  | Are there circumstances that prevent you from more frequent psychiatric/psychotherapeutic treatment of refugees or asylum seekers? | oe | - | s |  |
|  | How old are you? | ni | - | i |  |
|  | Gender | sc | Female  Male | n |  |
|  | Since when have you been working full-time in your current profession? | ni | - | n |  |
|  | Please enter the postal code of your main place of work | ni | - | n |  |

*Note*. mc – multiple choice; sc – single choice; ni – numeric input; oe – open-end; i – interval-scaled; r – rang-scaled; n – nominal-scaled; s – string.
